# A randomized controlled trial of an educational intervention to promote oral and dental health of patients with type 2 diabetes mellitus

**DOI:** 10.1186/s12889-020-8395-4

**Published:** 2020-03-04

**Authors:** Maryam Malekmahmoodi, Mohsen Shamsi, Nasrin Roozbahani, Rahmatollah Moradzadeh

**Affiliations:** 1 Department of Health Education and promotion, Faculty of Health, Arak University of Medical Sciences, Arak, Iran; 2 Department of Epidemiology, Faculty of Health, Arak University of Medical Sciences, Arak, Iran

**Keywords:** Type 2 diabetes mellitus, Health belief model, Oral hygiene, Diabetes care

## Abstract

**Background:**

Diabetes is the most prevalent disease resulted from metabolic disorders. This study aimed to investigate the effect of training based on health belief model (HBM) on oral hygiene-related behaviors in patients with type 2 diabetes mellitus.

**Methods:**

This study was conducted as an educational randomized controlled trial (single blind) on 120 patients with type 2 diabetes referring to a diabetes clinic selected through systematic sampling, who were assigned to two groups of control (*N* = 60) and intervention (*N* = 60). The data collection tool was a valid and reliable questionnaire based on HBM which was completed by both groups before the intervention. Then, the intervention group received 4 sessions of educational program based on HBM in 1 month, and the same questionnaire was completed again after 3 months and the data were analyzed through SPSS version 20 software with inferential statistics, t-test, paired t-tests, Chi square, Mann-Whitney test, and Wilcoxon test analysis.

**Results:**

Three months after the intervention, awareness of the patients and perceived susceptibility, benefits, self-efficacy, internal cue to action, and performance in oral and dental hygiene-related behaviors had a significant increase in the intervention group (*p* < 0.05). So that the performance of oral and dental hygiene in the intervention group increased from 2.16 ± 0.71 to 3.25 ± 0.49 (*p* = 0.001) after the education.

**Conclusion:**

Our results suggest that training patients with diabetes based on HBM as well as through active follow-up can enhance their skills in oral and dental hygiene-related behaviors. Controlling, monitoring and follow-up during the program are also recommended.

**Trial registration:**

Iranian Registry of Clinical Trials, IRCT 2017050733847N1. Prospectively registered 14 June 2017, http://en.irct.ir/trial/26011

## Background

Diabetes is one of the metabolic diseases and is a multifactorial disorder that is characterized by a chronic rise in blood sugar or hyperglycemia and is caused by either insulin secretion disorder or insulin dysfunction or both. Diabetes is also called a silent epidemic and a major public health problem and it accounts for 9% of all deaths worldwide [1]. Patients with uncontrolled diabetes have oral complications such as increased dry mouth and burning mouth. Alterations in collagen metabolism and consequently periodontal fiber changes, cause periodontal disease, which is due to the presence of microbial plaques and poor hygiene in most diabetics [2–4]. Salivary lactate levels in diabetics are higher than in healthy individuals and in advanced cases can reach up to 5 times the normal level, which is a contributing factor to caries [5]. Moreover diabetes mellitus affects virtually all tissues and organs of the body including the hard and soft tissues of the oral cavity, manifesting with several complications [6].

There are numerous studies showing that the prevalence, progression, severity, and extent of chronic oral diseases are significantly increased in diabetic patients. The main oral complications associated with diabetes, including infection of the gums, periodontal disease, tooth decay, dry mouth, bacterial infections and fungus, halitosis, and prolonged healing of wounds from dental treatments [7]. Belazi et al. in his research showed that the growth rate of Candida species was significantly higher in people with diabetes than in the healthy group [8].

Knowledge of diabetics about periodontal disease, dry mouth and prevention of oral and dental problems is of great importance in these patients [5, 9]. Patients with diabetes and their families need to learn and practice new lifestyle skills, including monitoring blood sugar, following medication instructions, having a proper diet, physical activity, and more. These skills are important both in controlling diabetes and in preventing or delaying its complications. Diabetic patients should be active participants in the educational process and in setting educational and behavioral goals [7, 10].

The only effective and efficient strategy for solving oral health problems is prevention and compliance with oral health [7]. According to the World Health Organization (WHO), health education is the best and most effective way of providing health care to people, both in terms of human resources and heavy cost of medical care [11].

According to WHO, education is the cornerstone of diabetes treatment. In fact, education has been recommended as an essential component of promoting good control of diabetes, and studies have shown that education is effective in controlling and treating the disease, and according to studies, proper training can reduce 80% of diabetes complications [11, 12]. The Health Belief Model is one of the oldest models of behavior analysis that has been used in numerous studies of health behaviors such as type 2 diabetes mellitus (T2DM) [13]. Social psychologists developed this model during the 1950s to predict the reasons for people’s unwillingness to engage in preventive health behaviors [14].

**Fig. 1:**
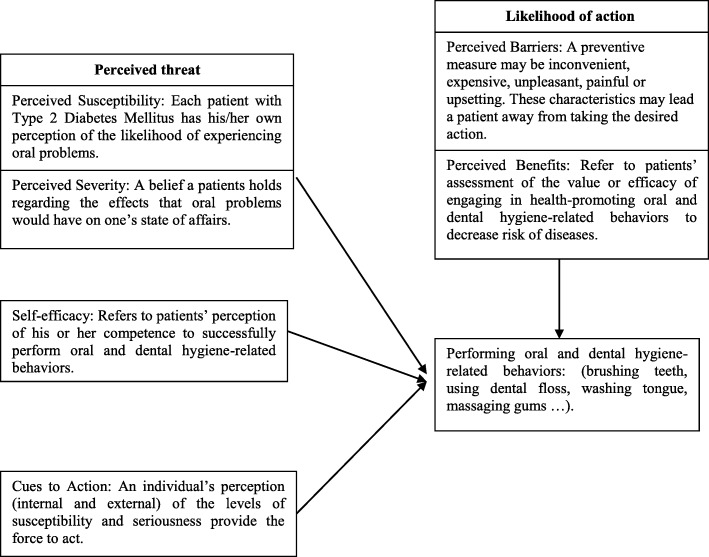
Health Belief Model (HBM). In this model, by developing perceived susceptibility and perceived severity to oral symptoms in diabetics, along with training on the perceived benefits, removing the perceived barriers, using internal and external cues to action as incentives for patients, and increasing their self-efficacy, oral performance and dental hygiene-related behaviors will be improved in these patients

In this model, diabetic patients first need to feel at risk for oral problems and to understand the seriousness of the complications. Then, in order to reduce these complications, they should understand the benefits of oral health care and reduce the barriers and move the patient toward oral health care through enhancing patients’ self-efficacy and empowerment in this regard as well as the impact of cues to action as internal and external incentives (Fig. 1).

Considering the lack of a theory-based study regarding the oral and dental hygiene in patients with diabetes, the current study was conducted to design and evaluate an educational intervention to promote oral and dental hygiene-related behaviors in patients with T2DM.

## Methods

### Study design

This study was conducted as an educational randomized controlled trial (single blind) on patients with diabetes referring to the county diabetes clinic in the city of Kashan, Iran from 2017 to 2018. From a total of 2500 diabetic patients referred to diabetic clinic, 120 patients who met the study’s inclusion criteria were randomly assigned into intervention and control groups (60 participants in each group).

According to the study by Baghiani Moghadam et al. [15] considering α = 5% and β =0.1.

Based on the following formula, the sample size was 58 patients in each convention and control group, which increased to 60 patients. Thus, the total number of samples was 120 patients.

$$ n=\frac{{\left({Z}_{1-\frac{\alpha }{2}}+{Z}_{1-\beta}\right)}^2\left({\delta}_1^2+{\delta}_2^2\right)}{{\left({\mu}_1-{\mu}_2\right)}^2} $$

In this study having means of 10.8 and 12.42 and standard deviations of 2.79 and 2.57 for the intervention group before and after the intervention respectively for perceived susceptibility construct in Baghiani Moghadam et al. [15], an effect rate of 0.6 was obtained indicating a large effect size and the same effect rate was considered for this study.

Of the patients who had medical records at the clinic, 120 were selected through systematic sampling and were randomly (every other person) assigned to the intervention (*n* = 60) and control (*n* = 60) groups through the rules of random allocation. Then the pre-test was administered to both groups based on the questionnaire. The intervention group received trainings based on HBM and the control group received routine cares. Then the patient were followed up for 3 months. After that the post-test was administrated and finally the effect of education on their oral and dental hygiene related behavior was re-evaluated. In this study the primary outcomes were constructs of HBM (perceived susceptibility, severity, benefits, barriers, cues to action, and self-efficacy) and secondary outcomes were oral and dental health behaviors.

According to Panel of Experts, 3 months of follow-up was considered sufficient time to establish consistency, stability, and sustainability in oral health care behaviors.

Inclusion criteria for the study included having medical records in the diabetes clinic, being between 40 and 60 years old, being literate, residing in Kashan, having no oral symptoms, having no history of radiotherapy and hemodialysis, having no other chronic systemic diseases, not taking any drugs that have side effects such as dry mouth, not wearing dentures, and signing an informed written consent. Exclusion criteria included having no desire to participate in the study, moving from Kashan to another city, not attending the training sessions regularly, and suffering from any other systemic disease.

**Fig. 2:**
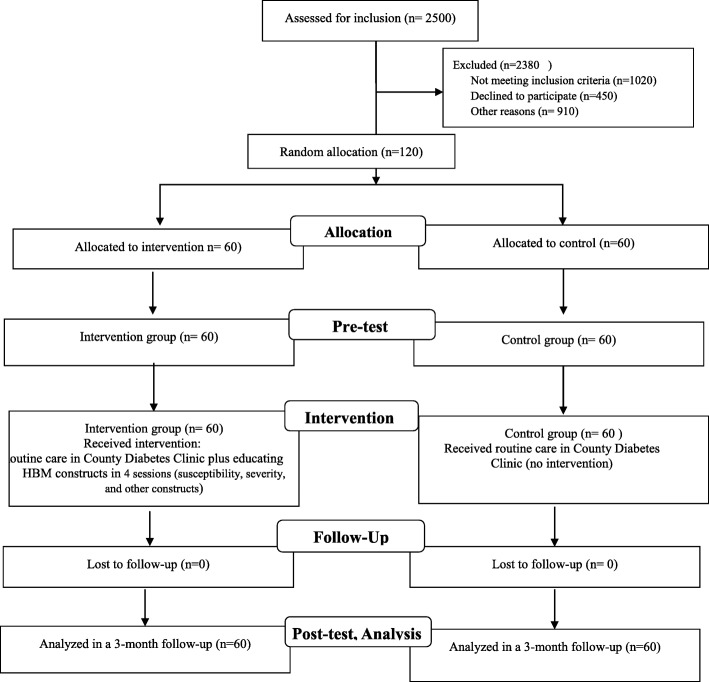
Flow diagram of the participants. From a total of 2500 diabetic patients referring to diabetes clinic, 120 were selected based on the inclusion criteria and then randomly divided into intervention and control groups (60 each). In both groups, pre-test was administrated and then the intervention group received HBM-based training and the control group received routine training. Three months later, post-test was administrated in both groups and oral health care behaviors were compared in both groups

The conceptual framework of the study was that according to the inclusion criteria, the samples referred to diabetes clinic were selected and then divided into intervention and control groups.. Then the pre-test was administered to both groups based on the questionnaire. After that the intervention group received trainings based on Health Belief Model. Using the perceived susceptibility construct, patients initially felt at risk of oral and dental problems and understood the complications and at the same time they were taught the benefits and barriers of preventive behaviors. Oral care-related behaviors were then taught using internal and external cues to action and increasing patients’ self-efficacy. The control group received routine diabetes clinic trainings. Then the patients were followed up for 3 months and then the post-test was administrated and the effect of education on their preventive behaviors was re-assessed. Figure 2 shows the study diagrams.

### Measures

The data collection tool in this study was a valid and reliable researcher-made questionnaire consisting of questions on demographic information, awareness, constructs of the Health Belief Model, and performance in oral hygiene-related behaviors in patients with T2DM. The validity and reliability of this questionnaire was approved and it was completed by both control and intervention groups before the educational intervention and 3 month after the educational intervention. In this tool, those questions with a Content Validity Ratio (CVR) greater than 0.62 and a Content Validity Index (CVI) greater than 0.79 were considered appropriate and were included in the study [16].

To confirm the reliability, the questionnaire was completed by 30 diabetic patients, and its reliability was 0.866 using Cronbach alpha. The validity of the questionnaire was also confirmed by three health education experts, three internal diseases specialists, an endocrinologist, a dentist, and an expert having a PhD in epidemiology and an executive focal point in the National Program for Prevention and Control of Diabetes in Iran after removing or modifying some of its statements.

The questionnaire of diabetic patients’ awareness of oral care consisted of 9 questions. Constructs of HBM questionnaire of oral hygiene-related behaviors consisted of perceived susceptibility (7 questions), perceived severity (10 questions), perceived barriers (7 questions), perceived benefits (8 questions), self-efficacy (11 questions), internal cues to action (triggers) (4 questions), and external cues to action (5 questions). The questionnaire of performance on oral hygiene-related behaviors also consisted of 10 questions.

### Scoring

In the awareness questions section, for each correct answer, a score of 1 and for each false answer, a score of 0 was considered, and the total score of the awareness section was calculated based on score 9. The Health Belief Model constructs questions were scored on a five-point Likert scale, with the answers being “strongly agree, agree, no idea, disagree and strongly disagree” from 1 to 5, respectively. Therefore the scores range of each model construct was finally calculated and reported between one and five.

The questions of performance questionnaire were scored on a 5-point Likert scale of behavior evaluation with the answers being “never, rarely, sometimes, often and always” from 0 to 4.

In this study higher score indicate higher level of awareness, perceived susceptibility, perceived severity, perceived benefits, perceived barriers, cues to action, self-efficacy, and performance of oral hygiene in diabetic patients.

### Intervention

Before performing the educational intervention and in pre-test step, the questionnaires were completed by both groups and entered the computer to determine patients’ educational needs and to determine the need for training of different structures in educational sessions.

Then, according to Health Belief Model and based on the results of the need assessment, the training program was prepared for four 120-min sessions in 1 month targeted at the intervention group. The materials were presented in the sessions through lectures, question and answer, Power Point slides presentation, and leaflets and booklets were provided for easier access of patients to educational resources during the study.

In the first training intervention sessions, the awareness of diabetic patients was emphasized with the aim of achieving better knowledge of diabetes and factors affecting deterioration and acceleration of oral complications. The second session’s focus was on perceived susceptibility and severity was touched by presenting the statistics on prevalence of oral problems resulted from diabetes and vulnerability of patients and severity of oral complications resulted from inappropriate blood sugar control and not performing oral hygiene-related behaviors. The third session’s focus was on perceived benefits, perceived barriers, and cues to action. The materials of this session emphasized on the benefits resulted from performing oral hygiene-related behaviors (reduced oral complications, decreased visit to the dentist and lower medical expenses, feeling the inner peace and joy), identifying and removing perceived barriers through performing oral hygiene-related behaviors (unawareness, physical weakness, fatigue, feeling bored, etc.), external cues to action affecting performing oral hygiene-related behaviors (including physicians, diabetes clinic nurses, family members, television, books and magazines in health centers, other diabetic patients), and the role of internal cues to action or triggers (motivation and inner peace resulted from performing hygiene-related behaviors).

The fourth session’s focus was on perceived self-efficacy and performance of oral and dental health hygiene behaviors. Self-efficacy construct was emphasized by empowering the patients to facilitate oral health care through presenting educational images on PowerPoint slides, practical training, distributing packages containing a toothbrush, a toothpaste and a dental floss among the patients, and providing them with booklets and leaflets. In the performance section the following behaviors were taught to the patients: brushing teeth, using dental floss, washing tongue, massaging gums, performing preventive behaviors and being aware of the possible oral complications of diabetes.

In this study, the control group received only routine care which included a monthly visit by a doctor, public health educators, a dietitian, and a nurse for less than 20 to 30 min at a clinic.

Three months after the educational intervention, the questionnaires were again completed by both groups.

### Statistical analysis

The data were analyzed using SPSS version 20 through descriptive statistics (Mean, SD, frequency, and percent) and inferential statistics (including independent t-test, paired t-test, Chi-square). To investigate the normality of the data, Kolmogorov-Smirnov test was used and normal distribution of the data was obtained. Concerning the gender difference between diabetic men and women with regard to oral hygiene behaviors (which the reviewer considered), and also due to the small size of the groups (men and women), the distribution of data was non normal and therefore nonparametric tests (Wilcoxon and Mann-Whitney) were used.

## Results

The average age of the diabetic patients in the control and intervention groups was 53.26 ± 4.46 and 53.48 ± 4.38 years, respectively, which showed no significant difference based on the results of the independent t-test (*p* = 0.675). Table 1 shows the other demographic characteristics of the patients (Table 1).

**Table 1:** Comparison of the intervention and control groups, concerning the demographic variables

Group Variable		Control		Intervention		***P***- Value
		Frequency (N)	Percent (%)	Frequency (N)	Percent (%)	
Sex	Female	36	60	40	66.6	0.44
	Male	24	40	20	33.4	
Health insurance	Yes	59	98.2	60	100	0.315
	No	1	1.8	0	0	
Marital status	Married	58	96.6	59	98.3	0.390
	Single	2	3.4	1	1.7	
Education level	Elementary school	35	58.4	43	71.6	0.019
	School diploma	18	30	16	26.6	
	College degree	7	11.6	1	1.8	
Income level	low	11	18.3	6	10	0.264
	middle	42	0.70	51	85	
	high	7	11.7	3	5	

The results showed that there was no significant difference between the intervention and control groups in terms of Health Belief Model constructs before the intervention. After the educational intervention, independent t-test showed a significant difference in terms of awareness, perceived susceptibility, perceived severity, perceived benefits and barriers, self-efficacy and internal cues to action between the intervention and control groups. So that the performance of oral and dental hygiene in the intervention group increased from 2.16 ± 0.71 to 3.25 ± 0.49 (*p* = 0.001) after the education.

However, there was no significant difference between the two groups in terms of external cues to action (Table 2).

**Table 2:** Comparison of the intervention and control groups, concerning the HBM, before and after the intervention

Group Variable		Control		Intervention		***P***-value^**a**^
		Mean	SD	Mean	SD	
**Knowledge**	**Before**	5.26	2.03	5.45	2.2	0.99
	**After**	5.85	1.5	8.16	0.95	0.012
	***P*-value**^**b**^	0.63		0.001		
**Perceived susceptibility**	**Before**	3.23	0.76	3.33	0.66	0.168
	**After**	3.64	1.01	4.09	0.49	0.156
	***P*-value**^**b**^	0.074		0.001		
**Perceived severity**	**Before**	4.22	0.61	4.33	0.68	0.71
	**After**	4.52	0.6	4.95	0.6	0.001
	***P*-value**^**b**^	0.17		0.001		
**Perceived barrier**	**Before**	3.38	0.75	3.54	0.74	0.68
	**After**	3.35	0.51	2.69	1.05	0.001
	***P*-value**^**b**^	0.77		0.001		
**Perceived benefit**	**Before**	4.29	1.13	4.18	0.66	0.94
	***P*-value**^**b**^	4.14	0.68	4.84	0.29	0.001
	**After**	0.72		0.001		
**Self-efficacy**	**Before**	3.37	0.71	3.42	0.62	0.65
	**After**	3.65	0.66	4.08	0.56	0.001
	***P*-value**^**b**^	0.04		0.001		
**Internal cues to action**	**Before**	3.92	0.85	4	0.74	0.60
	**After**	4	0.65	4.25	0.72	0.17
	***P*-value**^**b**^	0.27		0.033		
**External cues to action**	**Before**	3.67	0.70	3.50	0.83	0.67
	**After**	3.53	0.46	3.77	0.68	0.005
	***P*-value**^**b**^	0.71		0.33		
**Performance**	**Before**	2.28	0.76	2.16	0.71	0.97
	**After**	2.66	0.56	3.25	0.49	0.001
	***P*-value**^**b**^	0.714		0.001		

^a^Independent t test

^b^Paired t test

Concerning the gender difference between diabetic men and women with regard to oral hygiene behaviors, the results showed that in the intervention group, the behavior score of the diabetic women significantly increased from 2.22 ± 0.52 to 3.19 ± 0.58 (*p* = 0.025) after the intervention, while it was not significant in the male group (*p* > 0.05). The results have been presented in Table 3.

**Table 3:** Comparison of the intervention and control groups, concerning the performance of oral hygiene based on sex, before and after the intervention

Group Sex		Intervention N = 60 (Female = 40, Male = 20)		***P***-value^**a**^	Control N = 60 (Female = 36, Male = 24)		***P***-value^**a**^
		Before intervention	After intervention		Before intervention	After intervention	
**sex**	**Female**	2.22 ± 0.52	3.19 ± 0.58	0.025	2.35 ± 0.50	2.39 ± 0.58	0.18
	**Male**	2.40 ± 0.54	2.88 ± 0.65	0.42	2.17 ± 0.55	2.30 ± 0.61	0.36
	***P*-value**^**b**^	0.78	0.89		0.99	0.88	

^a^Wilcoxon test

^b^Mann-Whitney test

## Discussion

Based on the results of this study, the training intervention resulted in a significant enhancement in oral hygiene-related behaviors in the intervention group compared to the control group. In fact, this enhanced behavior can be attributed to the training method based on Health Belief Model. The educational method used can lead to positive attitudes to oral health in diabetic patients.

The significant change in awareness after the intervention in the intervention group showed the effect of the training intervention on enhancing the oral hygiene information related to oral hygiene in diabetic patients. These results were compatible with the results of many interventional studies such as those by Shabibi et al. [17] and Tawfik [18] on the awareness of diabetes care. Also, the results of other studies are compatible with those of the present study [14, 19–23]. Therefore, preparing trainings, educational texts and messages suitable for the audience features is one of the necessary principles of any training program as this study tried to present the training materials in a simplified way considering the age and education level of the participants.

In this study, perceived susceptibility of diabetic patients increased after the educational intervention, while the average score of perceived susceptibility did not changed in the control group. This increase can be attributed to the training classes, question and answer sessions, and group discussions aiming to sensitize the participants. This finding is compatible with those of Farahani et al. [24] on following the medication regimen in diabetic patients and other studies on nutritional and care taking behaviors of diabetic patients [25, 26].

The results of this study indicate an increased perceived severity among the patients comparing to before the intervention, which is due to the followings: the effect of training classes, warning about complications of the disease, presenting images on Power Point slides, distributing leaflets, and more interactions of the patients as a result of group discussions. However, this increase was not observed among the patients in the control group. This finding is compatible with the results of the studies on enhanced perceived severity among diabetic patients and caring aspects of nutrition and medication regimen and other caring behavior in diabetes control [24–28].

In this study, the perceived barriers of the diabetic patients decreased compared to those before the intervention. Factors such as insufficient awareness of different kinds of oral healthcare and the way to do them, physical weakness and diseases due to diabetes, high expenses of dentistry services, and being afraid of dental treatments were identified as the barriers and decreased through the educational intervention, providing standard and programed solutions and strategies aiming at enhancing the oral hygiene, provision of tools required for oral hygiene and teaching the necessary skills to the patients. The results were also compatible with those of other similar studies on the barriers perceived by diabetic patients regarding the diabetes healthcare [18, 24, 25, 28, 29].

The perceived benefits of patients increased after the educational intervention as they understood how observing oral hygiene can decrease the risk of tooth decay, cardiac and digestive diseases and halitosis, and can keep the gums healthy and reduce the dentistry expenses. This finding was compatible with the similar interventional studies such as those by Sohrabivafa et al. [19], Sharifirad et al. [25] and other studies [18, 24, 28, 29].

In this study an external cues to action in the intervention group slightly increased (from 3.5 to 3.77) after the intervention, which may be due to the fact that both groups have been receiving their information from social media and health centers and clinics before the intervention, and the educational intervention has not had much impact.

After the educational intervention self-efficacy construct increased by empowering the patients to facilitate oral health care through presenting educational images on PowerPoint slides, dividing the behaviors into smaller parts, practical trainings, distributing packages containing a toothbrush, a toothpaste and a dental floss among the patients, and providing them with booklets and leaflets. Self-efficacy increased the capability of diabetic patients for controlling and managing their oral hygiene and this behavior was kept and performed continuously after the educational intervention. This finding was compatible with those of Farahani et al. [24] and other studies on enhancing perceived self-efficacy of diabetic patients in diabetes cares [18, 25, 27–30].

The performance of oral hygiene-related behaviors by the patients increased in this study. This increase was due to preforming the oral hygiene-related behaviors (teaching the correct way of brushing teeth, using dental floss, washing tongue, massaging gums, performing preventive behaviors and being aware of the possible oral complications of diabetes).

In the present study, performing health behaviors was reported more in women than in men, so it seems that women are more sensitive to their health and the rate of their participation in health programs and their acceptance and practice of health behaviors are more than men. Therefore, designing educational programs to increase the participation of diabetic men in accepting oral health behaviors should be considered in future studies.

Successful control of oral health of diabetic patients mainly depends on performing hygiene-related behaviors by the patients [31]. In this study, the patients who stopped following the recommended oral hygiene-related behaviors mentioned lack of motivation, fatigue, laziness and high dentistry expenses as the reasons. Therefore, motivation and dentistry services with appropriate expenses are required for diabetic patients to perform the recommended oral hygiene-related behaviors correctly and continuously. The results of this study were compatible with those of the studies by Khani Jeihouni et al. investigating Health Belief Model constructs and tooth decay index in pregnant women [32], Friel et al. investigating the effect of oral hygiene intervention among teenagers in Ireland [33], and Shabibi et al. investigating the application of Health Belief Model to diabetic patients self-care [17].

With regard to the process of creating health behaviors in order to change people’s attitudes and creating stability and sustainability in health behaviors, group discussions with adequate time in educational sessions are needed. However, it is suggested that for the general population some of the educational material be provided indirectly (via media) through virtual booklets and leaflets with the aim of reducing the number of educational sessions.

One of the strengths of this study is that the educational intervention on oral hygiene care for diabetic patients was designed based on the needs assessment (pre-test) and programed according to Health Belief Model constructs and the patients’ behaviors were followed up 3 months after the educational intervention.

Some limitations of this study are using a small sample and self-reported questionnaires. Moreover the performance of diabetic patients in oral and dental hygiene related behavior over the past 3 months was assessed whereas longer follow-up could provide more accurate results.

It is suggested that in future studies the study design be based on double-blind randomization and it is also suggested that more studies be conducted with a larger sample size and in addition to the questionnaire, dental tests and examinations for diabetic patients be also used to assess health behaviors.

## Conclusion

The educational intervention based on Health Belief Model leads to increasing oral hygiene-related behaviors in patients with T2DM. It was found that diabetic patients’ skills about oral hygiene-related behaviors can be improved through training and continuous active follow-ups. This can ultimately reduce the incidence of oral complications in diabetic patients. It is recommended that controlling, monitoring and active follow-ups be also included in the training programs.

## Data Availability

The datasets generated during and analyzed during the current study are available from the corresponding author.

## Abbreviations

CVI: Content Validity Index
CVR: Content Validity Ratio
HBM: Health Belief Model
T2DM: Type 2 Diabetes Mellitus
